# Previous SARS-CoV-2 infections and their impact on the protection from reinfection during the Omicron BA.5 wave – a nested case-control study among vaccinated adults in Sweden

**DOI:** 10.1016/j.ijregi.2024.02.004

**Published:** 2024-02-28

**Authors:** Fredrik Kahn, Carl Bonander, Mahnaz Moghaddassi, Claus Bohn Christiansen, Louise Bennet, Ulf Malmqvist, Malin Inghammar, Jonas Björk

**Affiliations:** 1 Department of Clinical Sciences Lund, Section for Infection Medicine, Lund University, Lund, Sweden; 2 School of Public Health and Community Medicine, Institute of Medicine, University of Gothenburg, Gothenburg, Sweden; 3 Department of Clinical Sciences Malmö, Section for Social Medicine and Global Health, Lund University, Malmö, Sweden; 4 Department of Clinical Microbiology and Infection Prevention and Control, Skåne University Hospital, Lund, Sweden; 5 Department of Clinical Sciences Malmö, Section for Family Medicine, Lund University, Malmö, Sweden; 6 Clinical Studies Sweden, Forum South, Skåne University Hospital, Lund, Sweden; 7 Department of Laboratory Medicine, Division of Occupational and Environmental Medicine, Lund University, Lund, Sweden

**Keywords:** SARS-CoV-2 infection, Epidemiological surveillance, Variant of concern

## Abstract

**Objectives:**

We evaluated the protection afforded by SARS-CoV-2 infection-induced immunity against reinfection among working-age vaccinated individuals during a calendar period from June to December 2022 when Omicron BA.5 was the dominating subvariant in Scania County, Sweden.

**Methods:**

The study cohort (n = 71,592) mainly consisted of health care workers. We analyzed 4144 infected cases during the Omicron BA.5 dominance and 41,440 sex- and age-matched controls with conditional logistic regression.

**Results:**

The average protection against reinfection was marginal (16%, 95% confidence interval [CI] 7-23%) during the study period but substantially higher for recent infections. Recent infection (3-6 months) with Omicron BA.2 and BA.5 offered strong protection (86%, 95% CI 68-94% and 78%, 95% CI 69-84%), whereas more distant infection (6-12 months) with Omicron BA.1, BA.2, and the variants before Omicron offered marginal or no protection.

**Conclusions:**

These findings suggest that infection-induced immunity contributes to short-term population protection against infection with the subvariant BA.5 among working-age vaccinated individuals but wanes considerably with time, independent of the virus variant.

## Highlights

• Previous infection gave short-term protection against SARS-CoV-2 BA.5 infection.
• Recency of previous infection was more important than the virus variant for the protection.
• Waning of infection protection occurred independently of previous infection variant.

## Introduction

SARS-CoV-2 has emerged in multiple new strains, including B.1.1.7 (Alpha), B.1.351 (Beta), B.1.1.28.1 (Gamma), B.1.617.2 (Delta), and B.1.1.529 (Omicron). The Omicron subvariant BA.5 dominated for a long period also in countries with a high vaccine uptake and large groups with previous infection exposure. It is important to study infection-induced immunity in such populations to inform decision-makers within health care about the need and timing of additional booster doses and to guide the ongoing development of variant-specific vaccines [1]. Continued population surveillance is also warranted to rapidly detect if more severe variants begin to circulate. Previous studies have consistently shown that previous infection with early Omicron variants (BA.1 and BA.2) offer protection against reinfection with more recent variants (BA.4, BA.5, BA.2.75, and XBB) [2], [3], [4], [5], [6], [7], [8]. The estimated preventable fraction has in these studies varied between 50% and 90%, depending on the recency since the previous infection. Most previous studies have been hampered by short follow-up periods, which means that the effect of waning immunity has not been possible to separate reliably from the differential protection that different subvariants may have. Thus, there is a need for additional studies with longer follow-up to investigate the length of protection. The present study aimed to evaluate the protection afforded by SARS-CoV-2 infection-induced immunity against reinfection with the Omicron BA.5 subvariant among vaccinated individuals. The study was conducted in Scania County, southern Sweden, a region that had a rapid transition from early Omicron variants to BA.5.

## Methods

### Study design and data extraction

The overall study population included all persons residing in Scania County (Skåne), southern Sweden on December 27, 2020 (baseline) when the vaccinations started (n = 1,384,531) [9,10]. Data from national and regional register holders were linked using the personal identification number assigned to all Swedish residents [11]. Weekly updates on vaccination date, type of vaccine, and dose were obtained from the National Vaccination Register, and data on COVID-19 cases (defined by a positive SARS-CoV-2 test result) were from the electronic system, SMINet; both are kept at the Public Health Agency of Sweden. Regional health registers were used as complementary data sources to provide data on positive tests rapidly and to assess the comorbidities and disease outcomes.

Comorbidities were defined from diagnoses in inpatient or specialized care at any time point during the 5 years before baseline in the following disease groups (Supplementary Table 1): cardiovascular diseases, diabetes or obesity, kidney or liver diseases, respiratory diseases, neurological diseases, cancer or immunosuppressed states, and other conditions and diseases (Down syndrome, HIV, sickle cell anemia, drug addiction, thalassemia, or mental health disorder). The number of comorbidities in these groups was determined. Data from outpatient and general practitioner care settings were not available.

From the overall study population, we formed a cohort (n = 71,592; 73% females) restricted to working-age people (18-65 years old) who received their first vaccine dose relatively early (April 24, 2021 or sooner). With these restrictions, we expect the study cohort to mainly consist of health care workers because they were prioritized in the vaccination program (*Sensitivity analysis*). Health care workers were recommended to undergo testing throughout the study period in case of SARS-CoV-2 symptoms. The cohort was followed up longitudinally for positive SARS-CoV-2 tests until December 12, 2022 (week 49). Individuals who died or moved away from the region were censored on the date of death or relocation.

The follow-up period was grouped in accordance with routine sequencing of samples of infected cases in Scania County on the dominating variant of concern (VOC) (Supplementary Figure 1): (i) before Omicron (until 2021 week 47), (ii) transition to Omicron (2021 week 48-51), (iii) Omicron BA.1 dominance (60%; 2021 week 52 until 2022 week 1), (iv) transition to Omicron BA.2 (2022 week 2-3), (v) Omicron BA.2 dominance (89%; 2022 week 4-20), (vi) transition to Omicron BA.5 (2022 week 21-24), and (vii) Omicron BA.5 dominance (92%; 2022 week 25-49). In this study, we focused specifically on the follow-up period starting in 2022 week 25 (20 June) when Omicron BA.5 started to be the dominating VOC (>70%) and assessed the protective effect of the latest previous infection. An infection was only counted as a reinfection if it occurred more than 90 days after a previous infection. The time since previous infection was categorized as 3-6 months, 6-12 months, and at least 12 months. The strong association between dominating VOC and the time since previous infection during follow-up imposed restrictions on which combinations between variant and time that could be evaluated (Table 1). Although previous infections before Omicron all occurred at least 6 months ago, previous infections with BA.5 all occurred 3-6 months ago.

**Table 1:** Combinations of dominating virus variant at previous infection and time since previous infection that were available in data (marked with “X”) for the follow-up period 2022 week 25-49 with Omicron BA.5 dominance.

Dominating virus variant at previous infection	Time since previous infection, months		
	3-6	6-12	≥12
Before Omicron		X	X
Transition to Omicron		X	
Omicron BA.1	X	X	
Transition to Omicron BA.2	X	X	
Omicron BA.2	X	X	
Transition to Omicron BA.5	X		
Omicron BA.5	X		

### Statistical analysis

We used continuous density case-control sampling [12] nested within the study cohort. For each infected case during follow-up, 10 controls without a positive test the same week as the case or 90 days previous were randomly selected from the study cohort, matched with respect to sex and age (5-year groups). A control could only be sampled once within each matched set but could be sampled again into a different set. Using conditional logistic regression, we estimated the protection against infection with the Omicron BA.5 subvariant associated with type and time since previous infection, stratified according to the combinations that were available in data (Table 1) and with no previous infection as the reference group. In a secondary analysis, we estimated the additional protection from two previous infections. All analyses were adjusted for the number of vaccine doses received. Only vaccine doses obtained 7 or more days before the case date were counted in the analyses. BNT16b2 messenger RNA (Comirnaty, Pfizer-BioNTech) was the most frequently used vaccine type, with 68% of the 230,018 administrated doses in the study cohort. Positive protection was reported as 1 – odds ratio (OR), also referred to as the preventable fraction [6]. Negative protection for OR above one was calculated as 1 ÷ OR – 1 [13].

### Sensitivity analysis

Because early vaccination among working-age people in Sweden was not only offered to health care personnel but also to specific risk groups, we also conducted two sensitivity analyses to assess the robustness of the findings: (i) excluding individuals with organ or hematopoietic stem cell transplantation, undergoing dialysis, or with a third vaccine dose obtained early (September 28, 2021 or sooner; before third dose was offered to health care personnel) and (ii) updated case-control sampling after restricting the study cohort further to individuals without any comorbidity (n = 54,187).

## Results

A total of 4144 COVID cases occurred in the cohort during the study period with Omicron BA.5 dominance, which were used to sample 41,440 matched controls (Table 2). The cases and controls were similar with respect to civil status, proportion born abroad, and number of comorbidities. The vast majority of cases and controls had received at least one booster dose (93% and 88%, respectively). The risk of hospitalization in this cohort of vaccinated was low during the study period (1.4%, 56 of 4144 confirmed cases), albeit somewhat higher than during the period with BA.1 dominance in the same cohort (0.5%, 12 of 2501 cases).

**Table 2:** Characteristics of the COVID-19 cases (N = 4144) and sex- and age-matched controls (N = 41,440) during the follow-up period in June to December 2022 when Omicron BA.5 was the dominating SARS-CoV-2 subvariant in Scania County, Sweden.

		Cases, n (%)		Controls, n (%)
Total		4144 (100)		41,440 (100)
Age				
18-34		888 (21)		8815 (21)
35-44		859 (21)		8860 (21)
45-54		1138 (28)		11,135 (27)
55-65 years		1259 (30)		12,630 (30)
Sex				
Females		3387 (82)		33,870 (82)
Males		757 (18)		7570 (18)
Born abroad		691 (17)		8509 (20)
Civil status				
Married		1788 (43)		18,259 (44)
Divorced		542 (13)		5761 (14)
Single		1780 (43)		17,000 (41)
Widow/widower		34 (0.8)		420 (1.0)
Comorbidities				
0		3200 (77)		31,779 (77)
1		683 (17)		7151 (17)
≥2		261 (6.3)		2510 (6.1)
Vaccine doses				
1		18 (0.4)		243 (0.6)
2		280 (6.8)		4689 (11)
3		3198 (77)		30,187 (73)
4-5		648 (16)		6321 (15)
Previous SARS-CoV-2 infection	Months^a^	n(%)	Months^a^	n (%)
No	-	2622 (63)	-	24,428 (59)
Before Omicron	21	700 (17)	21	5758 (14)
Transition to Omicron	10	54 (1.3)	9.1	511 (1.2)
Omicron BA.1	8.9	152 (3.7)	8.8	1459 (3.5)
Transition to BA.2	9.2	215 (5.2)	8.4	2743 (6.6)
Omicron BA.2	8.9	390 (9.4)	7.5	6057 (15)
Transition to BA.5	5.5	5 (0.1)	5.5	87 (0.2)
Omicron BA.5	4.6	6 (0.1)	3.9	397 (1.0)

a Median time since previous infection in months.

**Table 3:** Odds ratio for SARS-CoV-2 infection in relation to previous infection type (model A) and additionally adjusted for time since previous infection (model B). Estimates obtained from conditional logistic regression with adjustment for number of vaccine doses (three to four vs two doses).

Previous SARS-CoV-2 infection	Model A		Model B	
	OR	95% CI	OR	95% CI
No previous infection	1.0	Ref.	1.0	Ref.
Before Omicron	1.15	1.03-1.28		
6-12 months			0.87	0.55-1.37
≥12 months			1.16	1.04-1.29
Transition to Omicron	1.04	0.77-1.40		
6-12 months			1.06	0.78-1.44
Omicron BA.1	1.01	0.84-1.23		
3-6 months			0.51	0.22-1.19
6-12 months			1.06	0.87-1.29
Transition to BA.2	0.75	0.64-0.88		
3-6 months			0.37	0.21-0.64
6-12 months			0.81	0.68-0.96
Omicron BA.2	0.60	0.53-0.68		
3-6 months			0.22	0.16-0.31
6-12 months			0.75	0.66-0.86
Transition to BA.5	0.51	0.21-1.25		
3-6 months			0.39	0.12-1.24
Omicron BA.5	0.13	0.06-0.30		
3-6 months			0.14	0.06-0.32

CI, confidence interval; OR, odds ratio.

**Figure 1:**
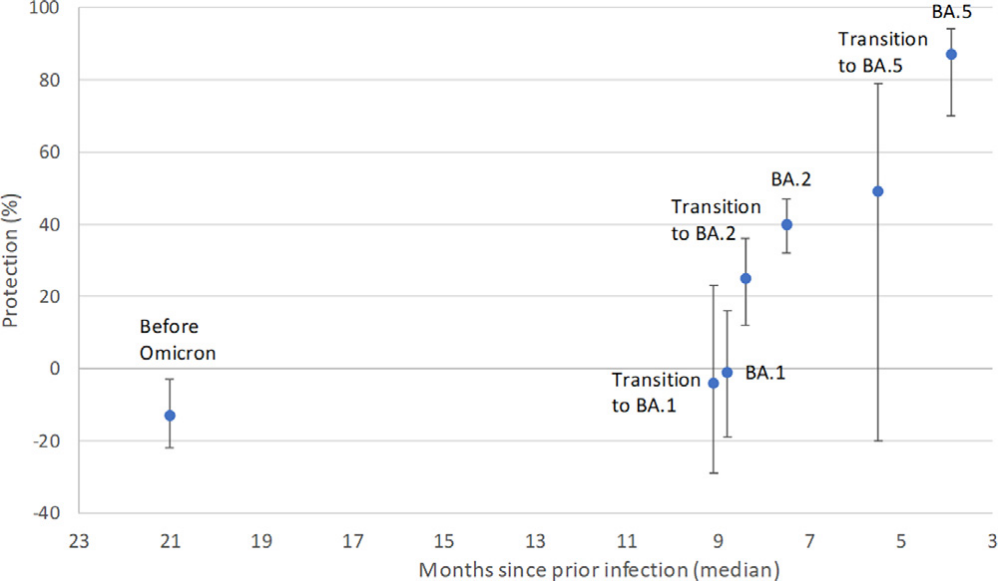
Estimated protection (%) against SARS-CoV-2 reinfection with the Omicron BA.5 subvariant in relation to average time since previous infection and dominating virus variant at the time of previous infection. Error bars represent 95% confidence intervals. Estimates obtained from conditional logistic regression with adjustment for number of vaccine doses (three to four vs two doses; model A in Table 3).

A previous SARS-CoV-2 infection was registered in 37% of the cases and 41% in the controls. The average protection against reinfection was marginal (16%, 95% confidence interval [CI] 7-23%) during the study period but substantially higher for recent infections. Although a clear protection associated with infection during the more recent periods with Omicron BA.2 and BA.5 dominance was observed, no remaining protection was observed for previous infections obtained before or during the Omicron BA.1 dominance (Figure 1 and Table 2, model A).

**Figure 2:**
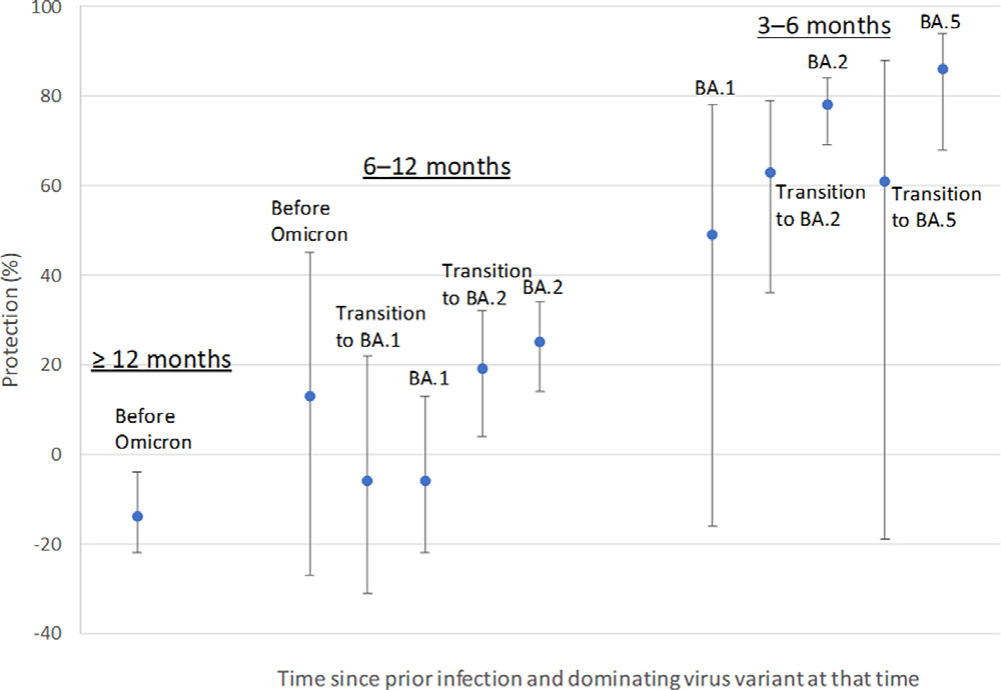
Estimated protection (%) against SARS-CoV-2 reinfection with the Omicron BA.5 subvariant in relation to time since previous infection (3-6 months, 6-12, and ≥12 months) and previous infection type. Error bars represent 95% confidence intervals. Estimates obtained from conditional logistic regression with adjustment for number of vaccine doses (three to four vs two doses; model B in Table 3).

A previous infection with more recent virus variants tended to have occurred more recently, on average, in the matched case-control sets (Table 2); however, there was, nevertheless, quite some overlap in the individual distribution of elapsed time (Supplementary Figure 2). After additional stratification of exposure for elapsed time, a previous infection of BA.2 or BA.5 offered strong protection after 3-6 months (78%, 95% CI 69-84% and 86%, 95% CI 68-94%) (Figure 2 and Table 2, model B). In the same time window, 3-6 months after previous infection, a weaker protection from BA.1 was suggested but with substantial statistical uncertainty (49%, 95% CI −16-78%). After 6-12 months, a marginal protection from BA.2 was observed (25%, 95% CI 14-34%), whereas BA.1 and the variants before Omicron offered no remaining protection in this time window. Likewise, no remaining protection from variants before Omicron remained beyond 12 months. Having two previous infections had no marked additional effect on protection (OR 0.88, 95% CI 0.70-1.1) (not in tables).

In the sensitivity analyses, excluding the risk group individuals who were vaccinated early (115 cases and 820 controls) only altered the associations marginally. The findings were also similar when we restricted the case-control sampling to individuals without comorbidities (3200 cases and 32,000 controls). As an example, the protection rates associated with previous BA.2 and BA.5 infection after 3-6 months were 76% (95% CI 66-84%) and 85% (95% CI 63-94%), respectively, in the restricted case-control analysis compared with 78 and 86% in the main analysis.

## Discussion

A salient finding of the present study among working-age vaccinated individuals was the relatively short-term protection against Omicron BA.5 reinfection, where recency of the previous infection was generally more important than the virus variant for the protection. A previous population study from Sweden with follow-up that ended before Omicron found that the relative risk of SARS-CoV-2 reinfection in individuals who survived a previous infection remained low for up to 20 months [14]. Long-lasting protection from previous infection before the emergence of Omicron have also been reported from several other populations [15], [16], [17], [18]. The emergence of Omicron has markedly shortened the duration of the protection [7,19]. Our study adds important new evidence in that respect by (i) having a long follow-up with Omicron BA.5 dominance, (ii) being able to stratify the infection-induced protection further by Omicron subvariants, and (iii) being able to stratify the analyses further for time since previous infection. Of the subvariants with overlapping recency in previous infection that were possible to compare in our stratified analysis (Table 1), recent infection (3-6 months) with Omicron BA.2 and BA.5 offered similarly strong protection, whereas more distant infection (6-12 months) with Omicron BA.1, BA.2, and the variants before Omicron offered marginal or no protection. A previous study from Portugal reported stability of immunity protection against BA.5 infection after infection with BA.1 or BA.2 subvariants over 8 months, after a relatively fast waning of protection initially similar to our study [19]. However, with longer follow-up and adjustments for differences in age and sex of the infected cases over time, we observed no plateau but rather a continuous decline in the immunity protection in our study.

We found a somewhat higher risk of hospitalization among confirmed cases during BA.5 than previously during the BA.1 dominance. A similar increase in risk of hospitalization was noted in a Danish study when contrasting BA.2 and BA.5 infections [4]; however, increasing selection in the confirmed cases during follow-up is an alternative explanation for these findings. It was not possible to assess the longitudinal changes in vaccine protection against hospitalization reliably in our low-risk cohort. A UK study found no evidence of decreasing vaccine effectiveness 2-14 weeks after a booster dose against hospitalization for BA.4 or BA.5 compared with BA.2 [20].

A previous immunological study among vaccinated individuals has suggested that the immune boosting by Omicron is lost with previous Wuhan-Hu-1 imprinting [21], whereas a recent epidemiological investigation among unvaccinated individuals showed additional protection from two previous infections: one non-Omicron and one Omicron [5]. We assessed the protection from two previous infections in a secondary analysis and saw similar protection in vaccinated individuals with previous Omicron infection, irrespective of whether they had another confirmed infection before Omicron.

The key strength of our study was the detailed individual-level data on vaccinations and infections during a long follow-up period. A major limitation was that we only had data on dominating virus variants at the population-level at different periods and not for individual cases. This may have led to bias toward the null from the nondifferential misclassification of previous infection exposure [22]; our study may, therefore, have underestimated the difference in infection-induced protection from different virus variants. Undetected current or previous infections owing to limited testing may also have biased the results, in particular, if a more recent infection leads to a lower probability of symptomatic infection or if adherence to the testing recommendations among the health care workers changed during follow-up. The uptake of booster doses was high in our cohort, and it was, therefore, not possible to differentiate the assessment of hybrid immunity, for example, in relation to adapted vaccines. The short periods of BA.1 and BA.2 dominance hampered the possibility to disentangle the general waning of immunity from differential protection across these subvariants. It should also be noted that even though our follow-up period was longer than in most previous studies, no person in the cohort had time since previous Omicron BA.5 infection exceeding 6 months or any previous Omicron infection at least 12 months ago. Therefore, continued monitoring of infection-induced protection associated with the various subvariants is warranted.

## Conclusion

Infection-induced immunity contributes to short-term population protection against infection with the subvariant BA.5 among vaccinated people of working age but wanes considerably with time, independent of the virus variant.

## Declarations of competing interest

The authors have no competing interest to declare.

## Appendix Supplementary materials

Image, application 1
