# Supplementary material for: Previous SARS-CoV-2 infections and their impact on the protection from reinfection during the Omicron BA.5 wave – a nested case-control study among vaccinated adults in Sweden
Source: IJID Reg. 2024 Feb 28;10:235–9. doi: 10.1016/j.ijregi.2024.02.004 (PMC10964055; doi:10.1016/j.ijregi.2024.02.004)

Previous SARS-CoV-2 infections and their impact on the protection from reinfection during the Omicron BA.5 wave – a nested case-control study among vaccinated adults in Sweden

Supplementary Material

**Supplementary Table 1.** Classification of comorbidities.

**Supplementary Figure 1.** Routine sequencing of samples of infected cases in Scania county, Sweden, 2021 – 2023.

**Supplementary Figure 2.** Months since prior infection in cases and controls stratified by dominating virus variant at the time of the prior infection.

**Supplementary Table 1.** Classification of comorbidities.

| **Disease group** | **ICD-10 codes (incl KVÅ-codes^a^)** |
| --- | --- |
| Cardiovascular diseases | I10-I15, I20-I25, I42-I43  I50, I60-I69  J81 |
| Diabetes or obesity | E10, E11, E66 |
| Kidney or liver diseases | K70.X, K74.3-K74.6, K75.4, K76.0  N18.5, N18.9  DR016, DR024 |
| Respiratory diseases | A15-A19  E84  I26, I27  J42, J43, J44, J45, J47, J84  J96, J98.2, J98.3 |
| Neurological diseases (including dementia) | G00-G99  F00-F03 |
| Cancer or immunosuppressed state (including organ transplantation) | C00-C99  KAS, FQA, FQB, JJC, GDG, JLE DR046, DR047, DR048  D80.0-D80.1  D80.5, D81, D82, D83 |
| Other conditions and diseases   - HIV - Thalassemia - Sickle cell - Mood disorders - Schizophrenia spectrum disorders - Substance use disorders - Downs syndrome | B20-B24  D56, D57  F10-F19, F30-F39, F20-F29  Q90 |

^a^ Swedish classification of certain interventions during health care visits

**Supplementary Figure 1.** Routine sequencing of samples of infected cases in Scania county, Sweden, 2021 – 2023. The dashed vertical lines show the grouping of the follow up period. The period with Omicron BA.5 dominance is 2022 week 25-49.

**Supplementary Figure 2.** Months since prior infection in cases and controls stratified by dominating virus variant at the time of the prior infection. Boxes represent median and quartiles in each time period, circles represent outliers exceeding 1.5 times the interquartile range.


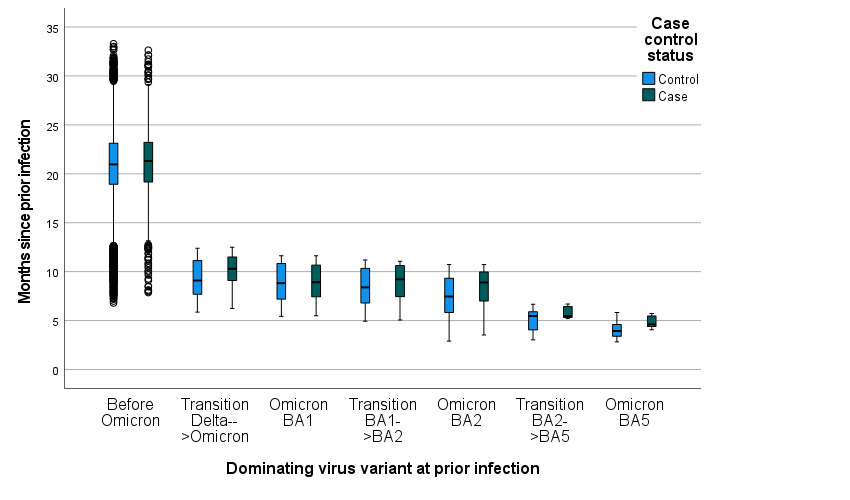

Supplement: Supplementary file 1 [file mmc1.docx]
